# Electron doping of exfoliated multilayer graphene induced by dissociative H_2_ adsorption due to long-term exposure to 80-bar H_2_ gas

**DOI:** 10.1080/14686996.2026.2627029

**Published:** 2026-02-05

**Authors:** Hyun-Seok Jang, Younghun Kim, Heewoo Lee, Soo Bong Choi, Jeongwoo Kim, Byung Hoon Kim

**Affiliations:** a Department of Physics, Incheon National University, Incheon, Republic of Korea; b Strategic Research Center for Smart Battery, Korea Basic Science Institute, Daejeon, Republic of Korea; c Intelligent Sensor Convergence Research Center, Incheon National University, Incheon, Republic of Korea; d Institute of Basic Science, Incheon National University, Incheon, Republic of Korea

**Keywords:** Multilayer graphene, high H_2_ pressure, dissociative H_2_ adsorption, hydrogen electron-doped graphene

## Abstract

Semiconducting graphene is expected to replace silicon in the electronics industry, and various methods have been proposed for this purpose. In this study, we demonstrate that the long-term exposure of multilayer graphene to 80 bar of molecular hydrogen induces electron doping in graphene. Ambipolarity behavior disappeared, and the current in the transfer curves decreased and increased in the negative gate voltage (*V*_*g*_) and positive *V*_*g*_ regions, respectively. The charge neutrality point shifted from 4.18 to over −80 V. Two resonant scatterings due to hydrogen adatoms were observed in the temperature-dependent transfer curves. For multilayer graphene with a boundary (edge), different behavior was observed in the transfer characteristics. Upon exposure to 80 bar of H_2_ pressure, the drain current of the time-dependent transfer curve rapidly decreased; however, it increased in the positive *V*_*g*_ region after 60 h of exposure to H_2_. Structural changes, particularly an increase in C‒H bonding, were observed using various characterization methods. These results were interpreted by the dissociative H_2_ adsorption of graphene. Molecular dynamics simulations also revealed the presence of electron doping due to dissociative adsorption. Furthermore, the simulations confirmed that dissociative adsorption occurred on the surface layer and at vacancies and defects.

## IMPACT STATEMENT

This study demonstrates significant electron doping in multilayer graphene through prolonged exposure to highpressure hydrogen gas, revealing a straightforward method for modifying its electronic properties without harmful chemicals.

## GRAPHICAL ABSTRACT

**Figure:**
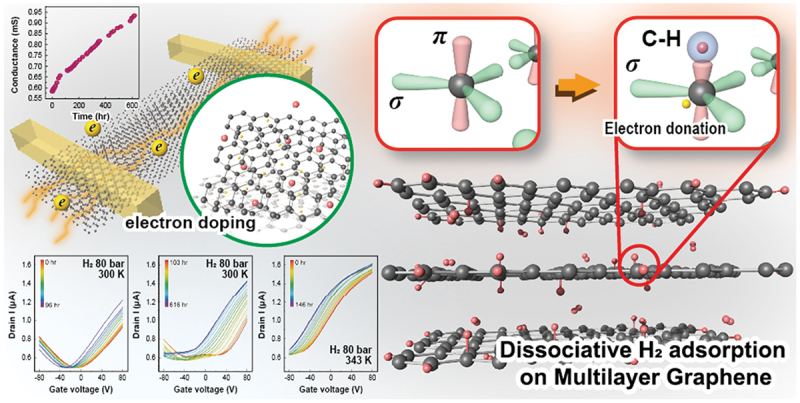

## 1. Introduction

Since the discovery of graphene, the electronics industry has regarded graphene as a substitute for silicon because of its remarkably high carrier mobility, transparency, high Young’s modulus, and chiral half-integer quantum Hall effect [1–7]. Graphene also exhibits superconductivity [8] and has been used in various fields, such as light-emitting diodes, supercapacitors, photocatalysts, mechanical transduction, sensors, wearable electronics, energy storage, energy harvesting, tissue engineering, gene delivery, and biomedical sensors [9–14]. Although graphene exhibits these exotic properties, controlling the carrier types (*n*- and *p*-types) of graphene must proceed to replace the silicon industry.

Various methods have been proposed to control the carrier types of graphene. *p*-type graphene has been induced by functionalization with pyrenebutyric acid [15], cationic nitrogen doping [16], nitric acid doping [17], and optical excitation in organic and inorganic sandwich structures [18]. *n*-type graphene can be obtained using various methods, such as nitrogen [19–23] and potassium [24] doping, KBr doping under deep-ultraviolet (UV) irradiation [25], and functionalization with lanthanide complex [26], polyvinylpyrrolidone [15], and ion gel films [27]. Other methods include coating [28] and doping [29] of organic materials, hybridization with MoS_2_ for *p-n* junction [30], heterostructure with carbon nanotubes [31] and ferroelectric LiNbO_3_ crystals [32], organic and inorganic sandwich structures under UV [18], and atomic-layer deposition with water [33] and ozone [34]. However, these methods require the use of harmful chemicals or complicated procedures to change the electronic structure of graphene.

Exposure to high-pressure gas is a possible route for modifying the electronic structure of materials. For example, the oxidation of V^4+^–V^5+^ of V_2_O_5_ nanowires due to a CO_2_ gas pressure of 45 bar has been reported [35]. High H_2_ pressure induces electronic structural changes in MoS_2_ [36–38], ZnO thin films [39], single-walled carbon nanotubes [40], and VO_2_ nanowires [41]. For graphene, the dissociative H_2_ adsorption has been considered a chemically unfavorable reaction. However, we previously reported that dissociative H_2_ adsorption resulted in the shift of the charge neutrality point (CNP) toward the negative gate voltage (*V*_*g*_) region due to 24-bar H_2_ gas pressure at 300 K, and this behavior was enhanced at 345 K [42]. A temperature-dependent (from 300 K to 340 K) CNP shift from −2.0 to −10.5 V during 10-bar H_2_ pressure was observed. The dissociative hydrogen atoms on multilayer graphene (MLG), resulting in C‒H bonds, were measured by quartz crystal microbalance [43]. In addition, the proof of *n*-type doping of graphene due to high H_2_ pressure has been reported [44–49]. However, the previous results mentioned above were achieved with relatively low pressure (maximum 24 bar H_2_ gas) and short time (~500 min). Thus, the CNP shift was small, and ambipolarity was still observed after exposure to H_2_.

In this study, we report the electron-doped MLG obtained by long-term exposure (762 h) to high-pressure H_2_ gas (80 bar) to provide fundamental evidence for the enhancement of dissociative H_2_ adsorption. The CNP shifted from 4.18 V in a vacuum to a negative *V*_*g*_ region and then vanished. The hole current was saturated. Finally, after 616 h, partially hydrogenated graphene (H-Gr) exhibited *n*-type semiconducting property under 80-bar H_2_ at 300 K. Based on the current-voltage (*I-V*) characteristics, the electrical conductance (*G*) of graphene increased from 0.55 to 1.5 mS. The temperature-dependent transfer curve of H-Gr confirmed a resonant scattering due to the presence of hydrogen adatoms acting as scattering centers. The structural change was investigated by Raman spectroscopy, X-ray photoelectron spectroscopy (XPS), Fourier transform infrared spectroscopy (FT-IR), atomic force microscopy (AFM), and X-ray diffraction (XRD). The ab initio molecular dynamics (MD) calculations, which demonstrated the dissociative H_2_ adsorption of the graphene.

## 2. Materials and methods

### 2.1. Sample preparation

Mechanically exfoliated MLG obtained from highly oriented pyrolytic graphite (HOPG) flake (HQ graphene, Netherlands) for charge transport property measurement was deposited on 300-nm SiO_2_/highly *p*-doped Si wafers (Figure S1a). The electrodes were patterned on the sample via e-beam lithography (JSM-6510, JEOL, Japan), followed by e-beam evaporation (TERA LEADER, Korea) of Cr/Au (5/50 nm). The sample was then wire-bonded to a PCB and loaded into the pressure chamber (Figure S1c). Structural changes were investigated using MLGs (Figure S2a – S2d) and bulk samples (Figure S2e – S2g) obtained from HOPG. The graphite powders (2–15 microns, 99,995%, Alfa Aesar, U.S.A.) were prepared for FT-IR measurements (Figure S2h).

### 2.2. Characteristics of structural change

The structural change of graphene induced by high-pressure gas exposure (after exposure to 80 bar H_2_ pressure at 343 K for 150 h) was measured using FT-IR (VERTEX 80 V, Bruker, U.S.A.), XRD (SmartLab, Rigaku, Japan) with Cu Kα radiation (λ = 1.5412 Å), XPS (PHI 5000 VersaProbe II, ULVAC-PHI, Japan), Raman spectroscopy (Raman-LTPL system, Witec alphy300, Witec, Germany) using 532-nm laser excitation, and AFM (XE-NSOM, Park systems, Korea).

### 2.3. Charge transport properties

*I-V* characteristics and transfer curves were measured using a semiconductor characterization system (4200-SCS, Keithley, U.S.A.) in a high-pressure stainless steel chamber. The devices were electrically characterized by a *V*_*g*_ sweep from −80 to 80 V with a drain-source bias of *V*_*ds*_ = 1.0 mV. First, the devices were loaded into the chamber, which was evacuated under 1.0 × 10^−6^ Torr at 300 K for 59 h and 325 K for 2 h. Second, the graphene sample was exposed to 99.999% H_2_ gas at pressures ranging from vacuum to 80 bar in 5-bar intervals at 300 K. We waited for 30 min at each interval to obtain a stable pressure in the chamber before the measurement. Third, 80-bar H_2_ pressure was maintained until *G* was saturated for 616 h. Fourth, H_2_ gas was released by 5-bar intervals, and the devices were then exposed to ambient air at 300 K. Fifth, the same procedure was performed at 343 K. The temperature-dependent transfer characteristics before and after exposure to 80-bar H_2_ gas pressure were measured in a cryostat (CH-202 10 K Cryocooler, Seongwoo Instruments, Korea).

### 2.4. Theoretical calculations

First-principles calculations were performed using *Quantum ESPRESSO* [50–52]. The Perdew–Burke–Ernzerhof functional of the generalized gradient approximation [53] was used to describe the exchange – correlation interactions among electrons, and Optimized Norm-Conserving Vanderbilt (ONCV) pseudopotentials [54] were used to describe the electron – ion interactions. The kinetic energy cutoff for the plane-wave basis set was set to 50 Ry. A self-consistent field convergence threshold of 10^− 6^ Ry was applied. MD simulations were performed using a time step of 1.0 fs at a temperature of 300 K. The radial distribution functions (RDFs) [55] were averaged over 0.2 ps. Brillouin zone integrations were sampled using 2 × 7 × 5 and 2 × 7 × 1 Monkhorst – Pack k-point grids [56] for stacked and monolayer graphene, respectively. A 4 × 2 rectangular supercell configuration under a hydrogen environment (16 atoms) was considered for hydrogenation simulation.

## 3. Results and discussion

### 3.1. Conductance change due to high H_2_ pressure

**Figure 1.**
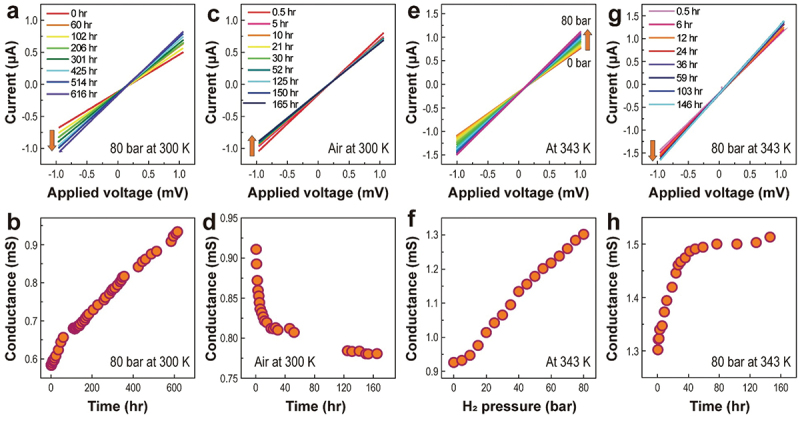
*I-V* characteristics and *G* of the MLG. The *G* was obtained from the *I-V* curve. (a) Time-dependent *I-V* curves and (b) *G* under 80 bar H_2_ gas pressure at 300 K. (c) Time-dependent *I-V* curves and (d) *G* in air at 300 K, showing that *G* nearly saturated after 120 h. (e) H_2_ pressure-dependent (from vacuum to 80 bar) *I-V* characteristics and (f) *G* at 343 K. (g) Time-dependent *I-V* curve and (h) *G* under 80-bar H_2_ gas pressure at 343 K.

Figure 1 shows the changes in *I-V* characteristics and *G* obtained from the linear fitting of *I-V* curves. The graphene was exposed to H_2_ gas up to 80-bar by 5-bar intervals at 300 K (Figure S3a). *G* changed from 0.553 mS in a vacuum to 0.584 mS at 80-bar H_2_ pressure. This value increased to 0.934 mS after 616 h in 80-bar H_2_ pressure at 300 K (Figure 1a,b). As the pressure was released, *G* decreased to 0.911 mS (Figure S3b). After exposure to air, *G* decreased due to water or oxygen molecules and then saturated to 0.780 mS after 165 h (Figure 1c,d). Interestingly, the saturated *G* was larger than the *G* value in a vacuum (0.553 mS). The increase in *G* due to H_2_ pressure can be interpreted as follows. Dissociative adsorption of hydrogen molecules on graphene breaks C = C double bonds, yielding two unpaired electrons. One of these unpaired electrons participates in the formation of a C – H bond, which disrupts the continuity of the π bond network, and the other electron is delocalized [57,58]. In addition, hydrogen adatoms donate electrons to the graphene [59]. Consequently, *G* increases [42]. Thermal energy accelerated this interaction. The graphene was loaded into the pressure chamber again, and the temperature of the chamber was then increased to 343 K in a high vacuum. The *G* value was recovered to 0.926 mS after 24 h in a vacuum, indicating the possibility of a reversible dissociative hydrogen adsorption process if the high temperature is applied. Figure 1e,fshows the dependence of *G* on H_2_ pressure at 343 K. The *G* increased from 0.926 mS in vacuum to 1.302 mS at 80 bar. As the exposure time increased at 80 bar, the *G* also increased up to 1.50 mS after 77 h and finally saturated until 146 h (Figure 1g,h). The effect of electron donation is clearly observed in the transfer characteristics because the additional electrons lead to *n*-type doping in graphene.

### 3.2. Transfer characteristics due to high H_2_ pressure

**Figure 2.**
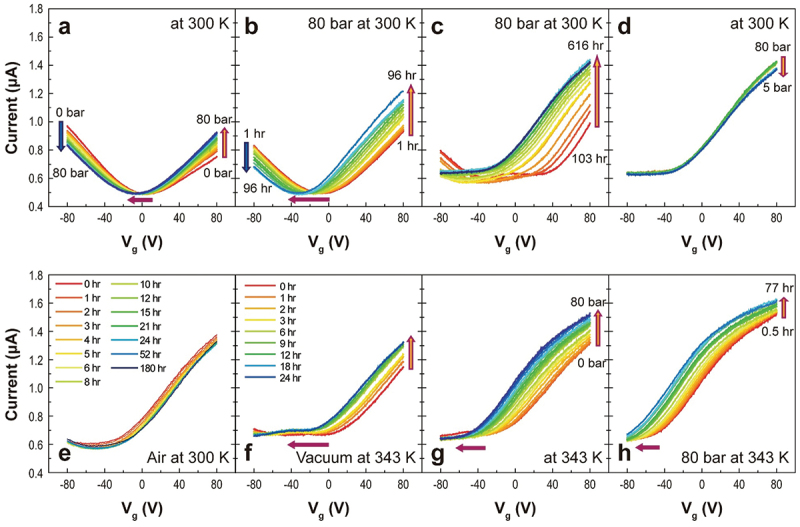
H_2_ pressure- and time-dependent transfer curves of the MLG. The red horizontal arrows represent the CNP (or threshold voltage) shift. (a) H_2_ pressure-dependent transfer curves at 300 K. (b) Time-dependent transfer curves from 1 to 96 h and (c) to 616 h under 80-bar H_2_ at 300 K. (d) Transfer curves during the release of H_2_ pressure at 300 K. (e) Time-dependent transfer curves under exposure to air at 300 K. (f) Time-dependent transfer curves in a high vacuum at 343 K from 0 to 24 h. (g) H_2_ pressure-dependent transfer curves from vacuum to 80-bar H_2_ at 343 K. (h) Time-dependent transfer curves under 80-bar H_2_ at 343 K.

Figure 2a displays the change in transfer characteristics as a function of H_2_ gas pressure up to 80 bar at 300 K. As the pressure increased, the currents in the positive and negative *V*_*g*_ regions increased and decreased, respectively. The CNP changed from 4.78 V in a vacuum to −6.61 V under 80-bar H_2_ pressure (red arrow). In addition, in the time-dependent transfer curves at 80 bar, the CNP significantly shifted to −32.27 V at 96 h (Figure 2b) and −69.35 V at 514 h (Figure 2c). Finally, the CNP vanished after 585 h, and the transfer curve with a threshold voltage exhibited *n*-type semiconducting behavior. The plateau observed after 585 h at 300 K (Figure 2c) can be interpreted by the quantum phase cancellation between multi-scattering paths due to the disorders caused by hydrogen adatoms.

The pressure in the chamber was released from 80 to 5 bar after exposure to H_2_ pressure for 616 h. The transfer curves were rarely changed (Figure 2d). Next, the sample was then exposed to air at 300 K. The broad CNP occurred again (Figure 2e). At a *V*_*g*_ value of 80 V, the current slightly decreased until 24 h; however, it increased after 52 h and was maintained until 180 h (Figure S4). Although the current level slightly decreases in the air, this result demonstrates that H-Gr remains stable in ambient conditions for at least 180 h. After the measurement of temperature-dependent transfer curves in a vacuum (1.0 × 10^−6^ Torr, it will be discussed later), the sample was reloaded into the pressure chamber. As the chamber was held in a vacuum at 1.0 × 10^−6^ Torr and 343 K for 24 h, the CNP shifted to the negative *V*_*g*_ region, and the current in the positive *V*_*g*_ region increased (Figure 2f). Upon exposure to H_2_ pressure at 343 K, the CNP shift and current increase were enhanced (Figure 2g). As the exposure time increased, the current was also augmented, and the CNP was shifted over −80 V, resulting in the realization of an electron-doped MLG using only H_2_ gas (Figure 2h; changes in the CNP and mobility are summarized in Figure S5). The *n*-type behavior was also determined by the current of the four characteristic conditions as a function of the carrier density, *n* (Figure S6a). After 77 h of exposure to 80 bar of H_2_ at 343 K, only the electron density existed and was 6 × 10^12^ cm^−2^ at *V*_*g*_ = 0 V (Figure S6b). Finally, high-pressure dependent electrical properties using one of the inert gases, He, were also measured to investigate the high-pressure effect (Figure S7). The result directly indicates that electron doping on MLG arises from the dissociative H_2_ adsorption rather than from pressure effects.

### 3.3. Temperature-dependent charge transport behavior

**Figure 3.**
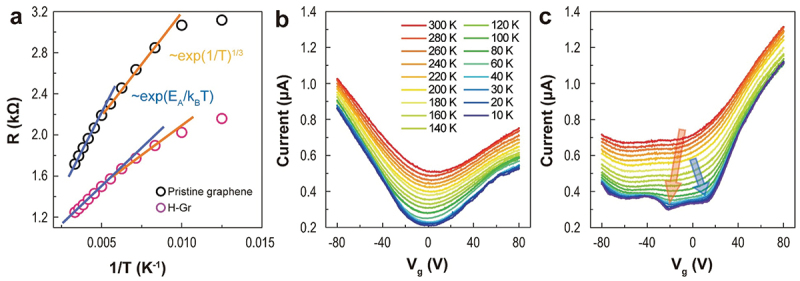
Temperature-dependent electrical transport properties of the MLG. (a) Temperature-dependent *R* at *V_g_* = 0 V, accompanied by fitting results using different charge transport models, including the arrhenius model and variable-range hopping. (b) Temperature-dependent transfer curves of pristine graphene. (c) Temperature-dependent transfer curves of H-Gr.

Figure 3a shows the temperature-dependent resistance (*R*) of before (black circles) and after (pink circles) H_2_ exposure measured at *V*_*g*_ = 0 V. The *R* of H-Gr is lower than that of pristine graphene. The *R* exhibited three distinct properties. First, the thermally activated process described by the Arrhenius expression at high temperatures (the blue dashed line), *R* = *R*_*0*_exp(*E*_*A*_*/k*_*B*_*T*), where *E*_*A*_ and *k*_*B*_ denote the activation energy and Boltzmann constant, respectively. Second, variable-range hopping in two-dimensional (2D) materials is expressed by *R* = *R*_*M*_exp(*T*_*M*_*/T*)^1/3^ in the middle temperature range. Third, saturation toward the Mott maximum resistance is observed at low temperatures (Figure S8). This behavior is similar to the trend previously reported on H-Gr [60]. The *E*_*A*_ values of pristine graphene and H-Gr are 12.3 and 9.51 meV, respectively. The small *E*_*A*_ of H-Gr indicates that charge carriers are more easily activated from the chemical potential to the energy associated with the percolation paths. The Mott temperature, *T*_*M*_, is inversely proportional to the density of states near the Fermi energy (*E*_*F*_). The small *T*_*M*_ of H-Gr (269.8 K) compared to that of pristine graphene (406.6 K) showed a large density of states near *E*_*F*_ of H-Gr, as the theoretical calculation for low-concentration hydrogen of H-Gr [61].

Low temperatures can rule out thermal energy. Thus, the temperature-dependent transfer curves were measured down to 10 K. For pristine graphene, the current decreased as the temperature decreased, as expected (Figure 3b). The CNP slightly shifted to a negative *V*_*g*_ region (from 4.78 V at 300 K to 0.804 V at 10 K), which was caused by the high vacuum (1.0 × 10^−6^ Torr). The temperature-dependent transfer curves of H-Gr were measured in a vacuum after 180 h of exposure to air (Figure S4). At 300 K, *n*-type characteristics and two broad peaks near −10 and −60 V were observed (Figure 3c). The peak at −60 V originated from the CNP shift, whereas the peak at −10 V resulted from the resonance scattering of the dimer, which was shifted to −20 V with decreasing temperature (red arrow). Another peak at 13.2 V emerged from 60 K and was visible at 10 K (blue arrow). This can be attributed to the resonance scattering of the *β* sublattice. The interpretation of the resonance scattering occurred due to hydrogen atom is as follows: Hydrogen adatoms (defects) create a localized state with narrow energy width near Dirac point, acting as the resonant scatterers. J. Katoch et al. observed two distinct resonant peaks appear in the *V*_*g*_-dependent resistance of H-Gr, akin to those shown in Figure 3c. They determined that both peaks are associated with hydrogen adatoms located on different graphene sublattices and also calculated the density of state for resonant defect levels of hydrogen. The energy level for the α sublattice (dimer site) is closer to CNP than that for the β sublattice (non-dimer site) [59]. This finding is consistent with the *V*_*g*_-dependent drain current observed in this study. The small fluctuation of current at low temperatures in both samples is caused by the interference of electrons scattered from a distribution of scattering centers [62]. These results indicate dissociative H_2_ adsorption behavior. The electrical properties of MLG provide evidence of dissociative H_2_ adsorption on MLG, resulting in electron doping that is further enhanced by prolonged exposure to high H_2_ pressure.

### 3.4. Structural change after H_2_ exposure

**Figure 4.**
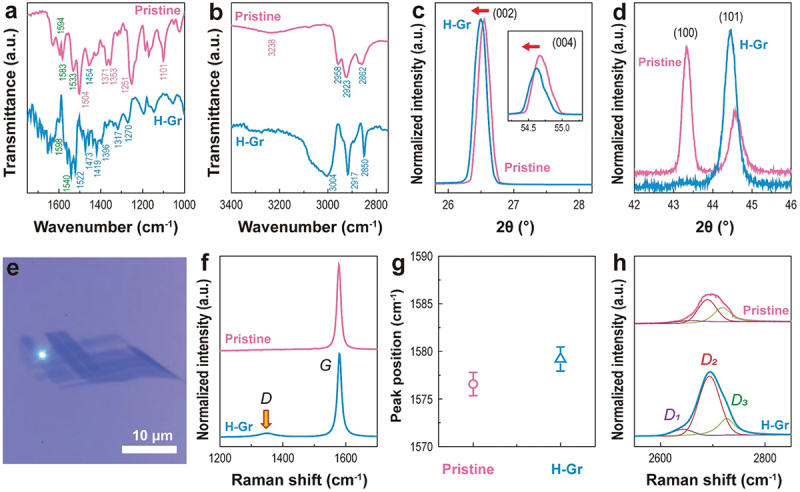
(a)-(b) FT-IR spectra of pristine (pink lines) and H-Gr (hydrogenated graphite powder, blue lines). (c)-(d) XRD patterns of pristine (pink lines) and hydrogenated HOPG (H-Gr, blue lines), showing diffraction peak shifts toward lower angles in H-Gr (red arrows). (e) Optical image of mechanically exfoliated MLG used for Raman spectroscopy. (f) Raman spectra in the *D-* and *G-* band regions for pristine (pink line) and hydrogenated MLG (H-Gr, blue line), obtained from the position marked in (e). (g) Peak positions of the averaged *G* band for pristine (pink circle) and hydrogenated MLGs (H-Gr, blue triangle), obtained from the eight points shown in Figure 4(e), Figure S9(a), and Figure S10. (h) Variation of *2D* band of pristine (pink line) and hydrogenated MLG (H-Gr, blue line), measured the position marked in (e).

Figure 4a,b shows the FT-IR results of graphite powder before and after exposure to 80 bar H_2_ gas. Before H_2_ exposure (Pristine), oxygen-related modes were observed at 1101 (C – O – C), 1251, 1353 (stretching mode of C – O), 1371 (bending of C – OH), and 1504 cm^−1^ (O – C – O bending vibration) (pink indices in Figure 4a). In contrast, in H-Gr, the modes at 1101, 1251, 1504 cm^−1^ disappeared, whereas those at 1353 and 1371 cm^−1^ weakened (blue line in Figure 4a). The C – H bending mode was observed at 1454 cm^−1^ in pristine graphite. However, the additional stretching and bending vibrations of C – H at 1270, 1317, 1396, 1419, 1473, and 1522 cm^−1^ were observed (blue indices) in H-Gr [63]. Vibration modes over 1530 cm^−1^ are relevant to C = C bonds. The modes at 1533 and 1540 cm^−1^ can be designated by *sp*^2^ C = C bonds. The peaks at 1600–1580 cm^−1^ in pristine graphite correspond to the stretching vibrations of the aromatic C = C bond. These modes were relatively weakened in H-Gr, indicating that some aromatic structures were broken due to H_2_ gas. The enhancement of C – H vibration was found in H-Gr at high wavenumbers (Figure 4b). In pristine graphite, symmetrical stretching in-phase C – H bonds (SSI, 2862 cm^−1^), asymmetrical stretching out-of-plane C – H bonds from methylene (ASOM, 2923 cm^−1^), and asymmetrical stretching out-of-plane C – H bonds from methyl (2958 cm^−1^) were observed [63]. An increase in the transmittance intensity of SSI and ASOM was shown in H-Gr. In addition, a new broad peak was observed at 3004 cm^−1^ in H-Gr. This mode corresponds to *sp*^*2*^ C – H bonds [63]. The O – H vibration near 3238 cm^−1^ in pristine graphite was indiscernible in H-Gr. According to the FT-IR results, upon H_2_ exposure, oxygen bonds with carbon atoms are lost, whereas C – H bonds are created and enhanced.

The (002), (004), (100), and (101) peaks are the representative planes in HOPG (Figure 4c,d). Shift to small angles occurred in (002), (004), and (101) peaks in H-Gr. The *d*-spacings increased from 3.356 to 3.363 Å for the (002) plane, from 1.678 to 1.680 Å for the (004) plane (Figure 4c), and from 2.031 to 2.037 Å for the (101) plane (Figure 4d). The structural changes are 0.21%, 0.12%, and 0.30% for the (002), (004), and (101) planes, respectively. The (100) peak was significantly reduced; however, the (101) peak became protuberant after exposure to H_2_ gas (Figure 4d). The symmetry of the (100) plane was weakened, whereas that of the (101) plane was relatively enhanced. This can be attributed to the energetically favorable resonance scattering of the *β* sublattice, i.e. the dissociative hydrogen adsorbs preferentially onto the edge and defect sites and the *β* sublattice related to the (100) plane [59].

To evaluate the structural impact of high-pressure hydrogen exposure, Raman spectra were collected from eight distinct locations across four samples. Figure 4e displays a representative optical image of the few-layer graphene used for this analysis. In pristine graphene, a sharp *G* band was observed at approximately 1576 cm^−1^. Following H_2_ exposure, a broad *D* peak emerged (Figure 4f and Figure S9), indicating the formation of *sp*^*3*^ defects. Additionally, the averaged position of the *G* peak exhibited a blue-shifted from 1576.4 ± 1.3 cm^−1^ in pristine graphene to 1579.2 ± 1.7 cm^−1^ in the H-Gr (Figure 4g). The second-order resonance (*2D* band) of pristine graphene is characterized by three constituent peaks located near 2640 (*D1*), 2690 (*D2*), and 2718 cm^−1^ (*D3*) (Figure 4h). While the *D1* peak was barely discernible in pristine graphene, this defect-originated peak became prominent in H-Gr. The *D2* and *D3* peaks correspond to 2- and 3-dimensional graphitic structures, respectively [64,65]. The intensification of the *D2* band of H-Gr is attributed to a reduction in the stacking order and an increase in the structural strain induced by the presence of hydrogen adatoms. Interestingly, while defect-related features were identified in the *2D* band of the MLG surface, the *D* band remained absent both before and after exposure to high-pressure H_2_ (Figure S10). This indicates that hydrogenation-induced defects are predominantly concentrated at domain boundaries and edges (Figure 4e and Figure S9).

XPS analyses of HOPG before and after exposure to H_2_ pressure were performed. The C1s peak was composed of *sp*^*2*^, *sp*^*3*^, C – O, C = O, and π–π* shake-up feature, which were attributed to the aromatic structure of the benzene ring (Figure S11a and S11b). The amounts of all species except the *sp*^*3*^ bonds, decreased in H-Gr. In other words, the amount of *sp*^*3*^ bonds increased from 31.26% to 36.19% (Table S1). This is due to an increase in the number of C – H bonds. The oxygen species (O – H, C – O, and C = O) in the O1s peak were also reduced after H_2_ exposure (Figure S11c and S11d, Table S1). Since *sp*^*3*^ bonding is considered a direct indicator for C‒H covalent bond formation, we focused on the 4.93% increase in *sp*^*3*^ bonds after exposure to H_2_. This corresponds with the obtained electron carrier density from the transfer curve, −5.52 × 10^12^ cm^−2^ (Figure S12). Although meaningful structural changes were observed in the FT-IR, Raman spectroscopy, and XPS results, the change in height obtained from AFM was small but highly consistent with the XRD results, showing an increase of 0.195–0.207% (Figure S13).

### 3.5. Comparison of dissociative H_2_ adsorption between edge and surface

**Figure 5.**
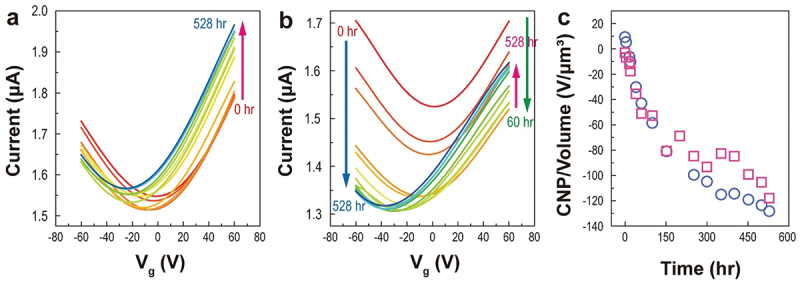
Time-dependent transfer curves of (a) s-MLG and (b) MLG-b at 300 K/80 bar. (c) Change in CNP per volume of s-MLG (pink squares) and MLG-b (blue circles).

At the edges and vacancy defects in graphite, the dissociative energy barrier of H_2_ is reduced [66–68], as observed in Raman spectroscopy. We prepared an MLG separated into two parts, one part has an even surface (s-MLG, blue circle in Figure S14a and S14b) and the other part has a boundary (white circle in Figure S14a and S14c), to compare the edge and surface effects of graphene and to confirm reproducibility of electron doping on MLG. A 2.17-nm height difference was observed in the MLG with a boundary (MLG-b) using AFM (Figure S14c). Under 80-bar H_2_ pressure, the time-dependent (from 0 h to 528 h) transfer characteristics of s-MLG at 300 K are similar to those shown in Figure 2 (Figure 5a). For MLG-b, a different behavior is observed. The drain current in the transfer curve decreased upon exposure to 80 bar of H_2_ until 60 h (green arrow in Figure 5b), indicating that gap-opening occurred rapidly because dissociative hydrogen atoms acting as defects were first adsorbed at the boundary (edge). After 60 h of exposure, the current due to electrons (the positive *V*_*g*_ region) monotonously increased (pink arrow). However, the hole current (blue arrow) continuously decreased until 352 h and then slightly increased, finally it was saturated. This is attributed to competition between the surplus electrons and attached hydrogen atoms on and into the MLG layers after dissociation. The surplus electrons had a dominant effect after 60 h. Figure 5c shows that the variation in CNP per volume of MLG-b from 0 to 528 h is larger (137.51 V/μm^3^) than that of s-MLG (114.93 V/μm^3^). This proves that the potential barrier for H_2_ dissociation is smaller at the edges than at the surface of graphene. The dissociative H_2_ adsorption was confirmed via an MD simulations.

### 3.6. Theoretical evidence for dissociative H_2_ adsorption

**Figure 6.**
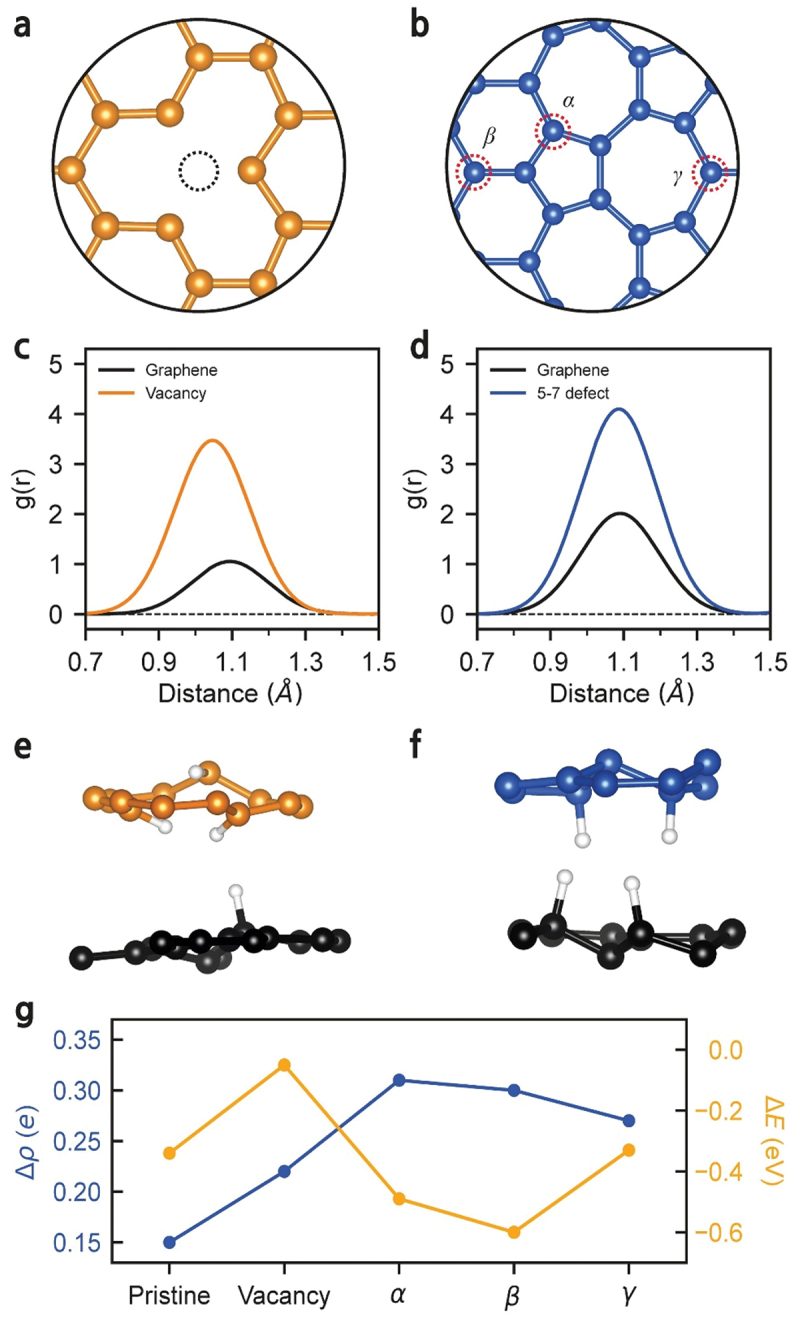
MD simulations of hydrogenated stacked graphene. Atomic structures of (a) single vacancy defect (orange) and (b) 5–7 defects (blue). Three distinct adsorption sites are represented as *α*, *β*, and *γ*. RDF of graphene containing (c) a vacancy or (d) 5–7 defects in a hydrogen environment. The atomic positions are averaged over 0.2 ps after hydrogen adsorption. (e)-(f) snapshots of hydrogenated graphene layers. Hydrogen adsorption at the defective layer (orange/blue) induces subsequent hydrogen adsorption in the adjacent pristine graphene layer (black). (g) Variation in charge density (blue) and average energy of occupied carbon states (orange) upon hydrogenation for pristine, vacancy, and 5–7 defect (*α-γ*) layers.

To investigate the adsorption behavior of hydrogen atoms on graphite, we performed *ab initio* MD simulations. Because pristine graphite is generally inert toward hydrogen adsorption, we considered representative defect structures commonly observed in graphite – specifically, a single vacancy (Figure 6a) and 5–7 defects (Figure 6b) – to simulate the dissociation of hydrogen molecules and the subsequent adsorption process. The calculated RDF reveals that hydrogen atoms are preferentially absorbed in the vicinity of these defect sites (Figure 6c,d). Remarkably, hydrogen adsorption was also observed on the pristine, defect = free graphene layer, as indicated by the black curves in Figure 6c,d. During the hydrogen dissociation process, one hydrogen atom from the molecule is attracted to the defect site, while the other forms a chemical bond with a neighboring graphene layers (Figure 6e,f and Movie S1, Movie S2). These observations demonstrate that, contrary to conventional expectation, a substantial number of hydrogen atoms were adsorbed not only at the defect sites but also on the pristine graphene layers.

The charge density variation and the average energy of occupied carbon states were also calculated to investigate charge transfer during the hydrogenation process (Figure 6g). In all structures considered – the pristine, the vacancy, and the 5–7 defect layers – carbon atoms gain electrons from adsorbed hydrogen atoms, confirming that hydrogenation results in *n*-type doping. Correspondingly, the average energy of the occupied carbon states shifts downward, consistent with charge transfer from hydrogen to carbon atoms during the hydrogenation process.

## 4. Conclusion

Upon long-term exposure of MLG to 80 bar of hydrogen molecules, electron doping on MLG was investigated using *I-V* and transfer characteristics. As the H_2_ pressure increased at 300 K, the *G* increased, and the current of the transfer curves decreased and increased in the negative *V*_*g*_ and positive *V*_*g*_ regions, respectively. The CNP also shifted to the negative *V*_*g*_ region. These behaviors were enhanced in the time-dependent transfer curves at a H_2_ pressure of 80 bar at 343 K, after which the CNP disappeared. At 300 K, the comparison of the time-dependent transfer curves between s-MLG and MLG-b at 80 bar of H_2_ shows that the potential barrier for H_2_ dissociation is smaller in MLG-b than in s-MLG. Two dips were observed in temperature-dependent transfer curves of H-Gr, resulting from the resonance scattering of the *β* sublattice due to hydrogen adatoms. The structural change of H-Gr was demonstrated using FT-IR, Raman spectroscopy, XPS, and XRD analyses. The dissociative adsorption of H_2_ molecules in MLG was also investigated using ab initio MD simulations, which were well consistent with the experimental results.

## Supplementary Material

Supplemental Material

Supplemental Material

Supplemental Material

## Data Availability

The data that support the findings of this study are available from the corresponding author upon reasonable request.
