# Supplementary material for: Electron doping of exfoliated multilayer graphene induced by dissociative H2 adsorption due to long-term exposure to 80-bar H2 gas
Source: Sci Technol Adv Mater. 2026 Feb 5;27(1):2627029. doi: 10.1080/14686996.2026.2627029 (PMC12943807; doi:10.1080/14686996.2026.2627029)
Supplement: Supplemental Material [file TSTA_A_2627029_SM0575.pdf]

Supporting Information

Electron doping of exfoliated multilayer graphene induced by dissociative H_2 adsorption due to long-term exposure to 80-bar H_2 gas

Hyun-Seok Jang[a,b,‡], Younghun Kim[a,‡], Heewoo Lee[a,c], Soo Bong Choi[a,c], Jeongwoo Kim[a,c,*], and Byung Hoon Kim[a,c,d,*]

[a] *Department of Physics, Incheon National University, Incheon, 22012, Republic of Korea*

[b] *Strategic Research Center for Smart Battery, Korea Basic Science Institute, Daejeon, 34133, Republic of Korea*

[c] *Intelligent Sensor Convergence Research Center, Incheon National University, Incheon 22012, Republic of Korea*

[d] *Institute of Basic Science, Incheon National University, Incheon 22012, Republic of Korea*

*Jeongwoo Kim - Department of Physics, Incheon National University, Incheon, 22012, Republic of Korea; Intelligent Sensor Convergence Research Center, Incheon National University, Incheon, 22012, Republic of Korea; orcid.org/0000-0002-4070-1878; E-mail: kjwlou@inu.ac.kr

*Byung Hoon Kim – Department of Physics, Incheon National University, Incheon, 22012, Republic of Korea; Intelligent Sensor Convergence Research Center, Incheon National University, Incheon, 22012, Republic of Korea; Institute of Basic Science, Incheon National University, Incheon, 22012, Republic of Korea; orcid.org/0000-0003-1118-8590; E-mail: kbh37@inu.ac.kr

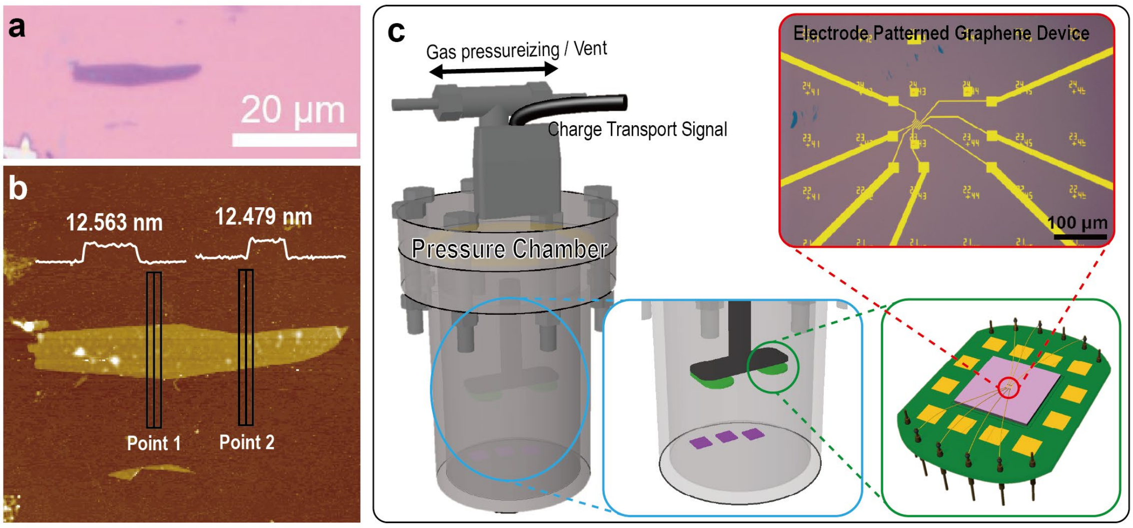

Figure S1. (a) Optical image of the multilayer graphene used for charge transport property characterization. (b) AFM topography of graphene and line profiles of point 1 and point 2. The heights of the two points were the averaged values. (c) Schematic diagrams and optical image of the pressure chamber and the prepared graphene device.

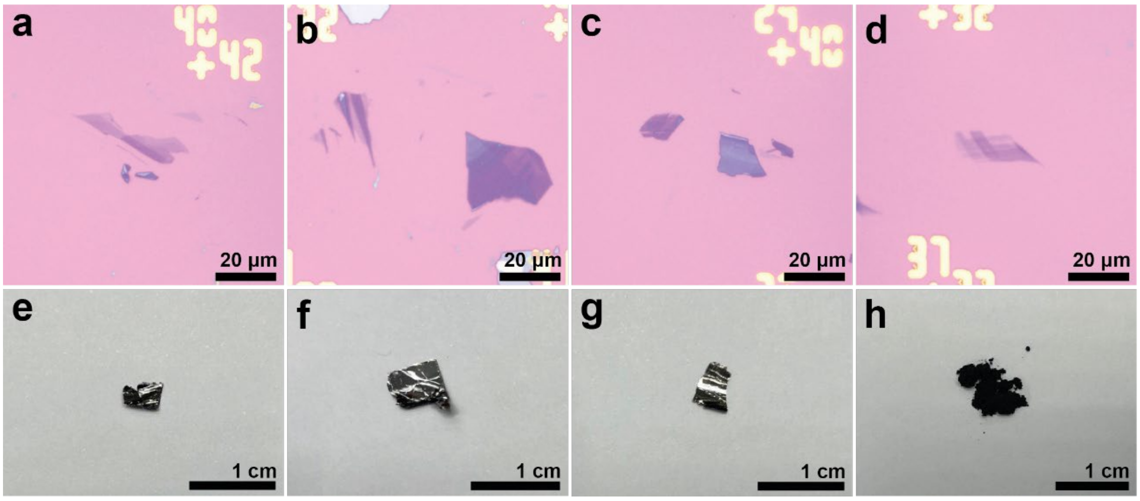

Figure S2. Samples for investigation of structural changes. (a)-(d) Mechanically exfoliated few-layer and multilayer graphene for Raman spectroscopy and AFM analysis. (e)-(g) HOPG for XRD and XPS. (h) Graphite powder for FT-IR

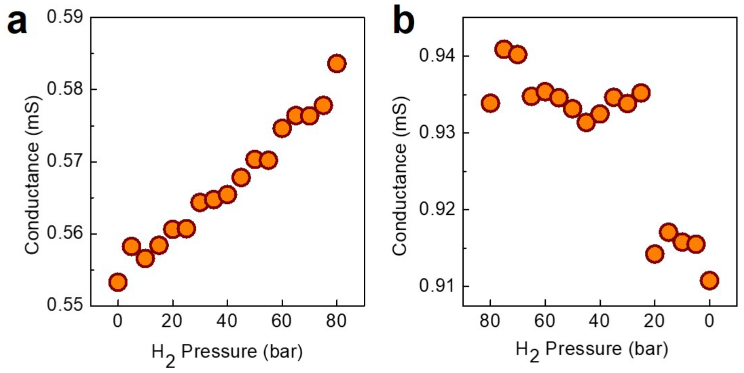

Figure S3. Variation in conductance due to H_2 pressure. H_2 pressure-dependent conductance when pressure (a) increased and (b) released.

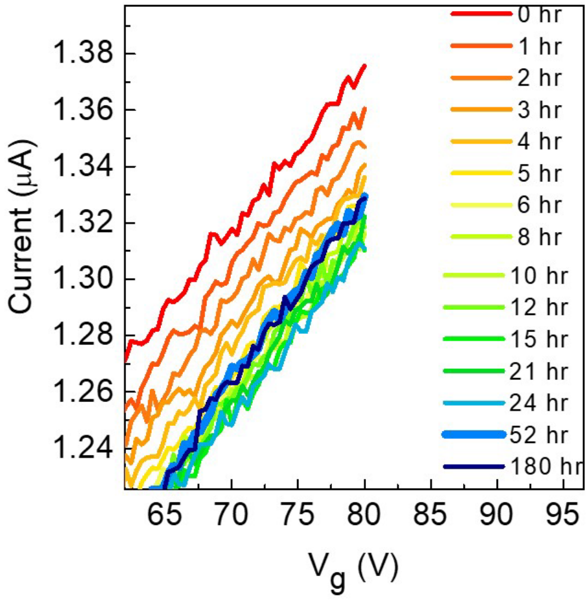

Figure S4. Transfer curves of graphene exposed to air at 300 K

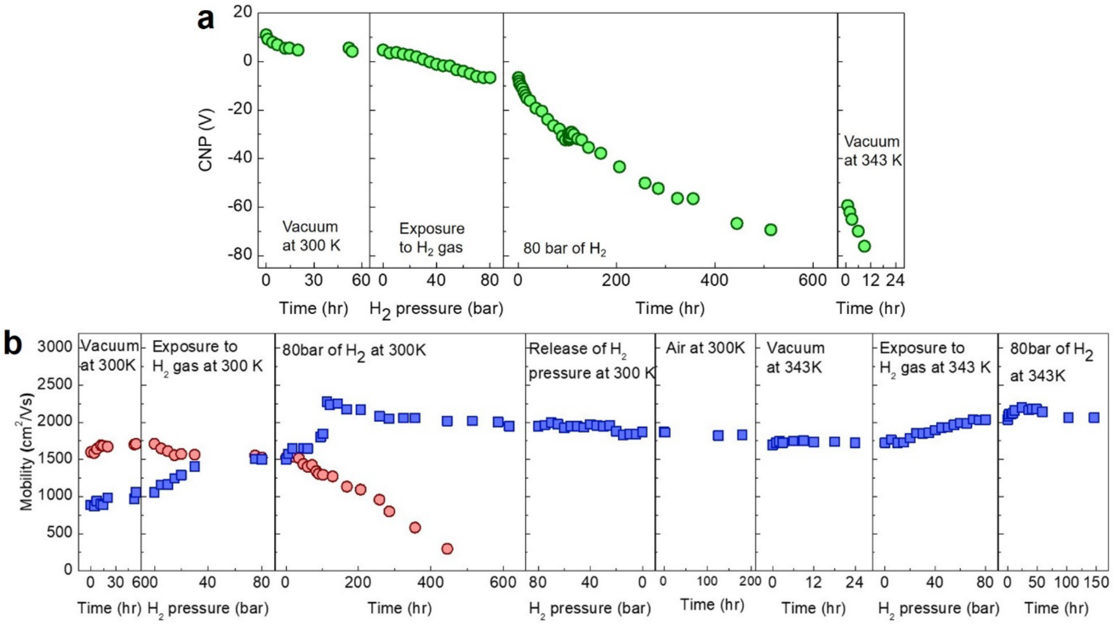

Figure S5. (a) Change in CNP. (b) Variations in electron (blue) and hole (dark pink) mobility

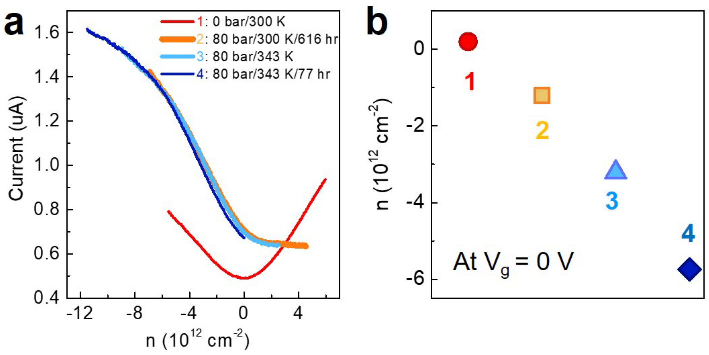

Figure S6. (a) Carrier density-dependent current under four different conditions. (b) Carrier densities at V_g = 0 V under each condition

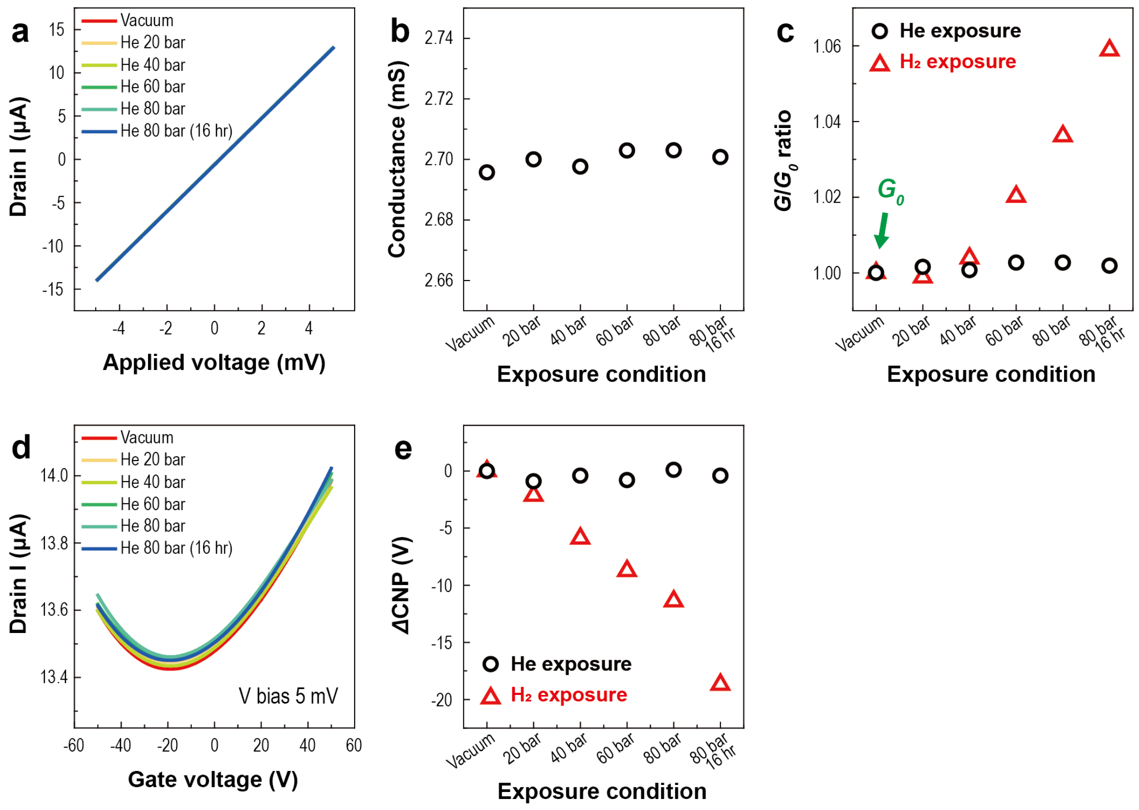

Figure S7. (a) He pressure-dependent *I-V* characteristics of graphene and (b) *G* obtained from *I-V* curves. (c) The variation G/G_0 of graphene during He (black circle) and H_2 (red triangle) exposure. (d) He pressure-dependent transfer characteristics of graphene and (e) shift of the CNP of graphene due to He (black circle) and H_2 (red triangle) exposure

As shown in Figure S7, the electrical properties were measured under the following exposure conditions: vacuum, He at 20 bar, He 40 bar, He 60 bar, and He 80 bar, and prolonged He exposure at 80 bar for 16 h. The *I-V* characteristics and the *G* remained unchanged throughout the exposure sequence (Figure S7(a) and (b)). The initial *G* under vacuum was 2.70 mS. In addition, the *G* ratio relative to vacuum (G/G_0) showed negligible variation compared to that observed under H_2 exposure (Figure S7(c)). The transfer characteristics were also measured under the same exposure conditions. As shown in Figure S7(d), the transfer curves rarely changed. In contrast to H_2 exposure (red triangle in Figure S7(e)), the CNP showed an indiscernible shift under He exposure (black circle in Figure S7(e)). These results directly indicate that electron doping on MLG observed in this study arises from the dissociative hydrogen adsorption on graphene rather than from pressure effects.

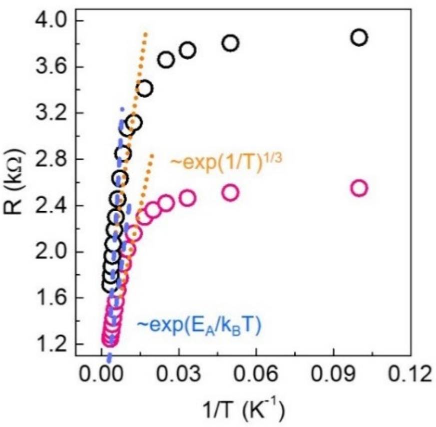

Figure S8. The electrical transport properties before (black circles) and after (pink circles) H_2 exposure

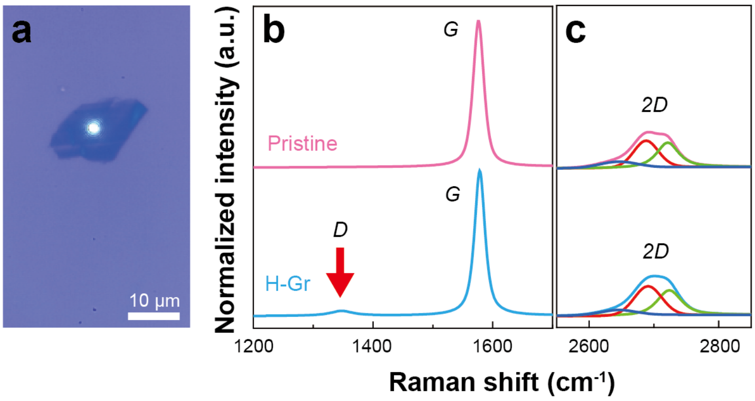

Figure S9. (a) Optical image of laser position for Raman study at multilayer graphene with multiple boundaries and edges. (b) *G* band and (c) *2D* band before and after exposure to H_2. The red arrow indicates the development of the *D* band in H-Gr

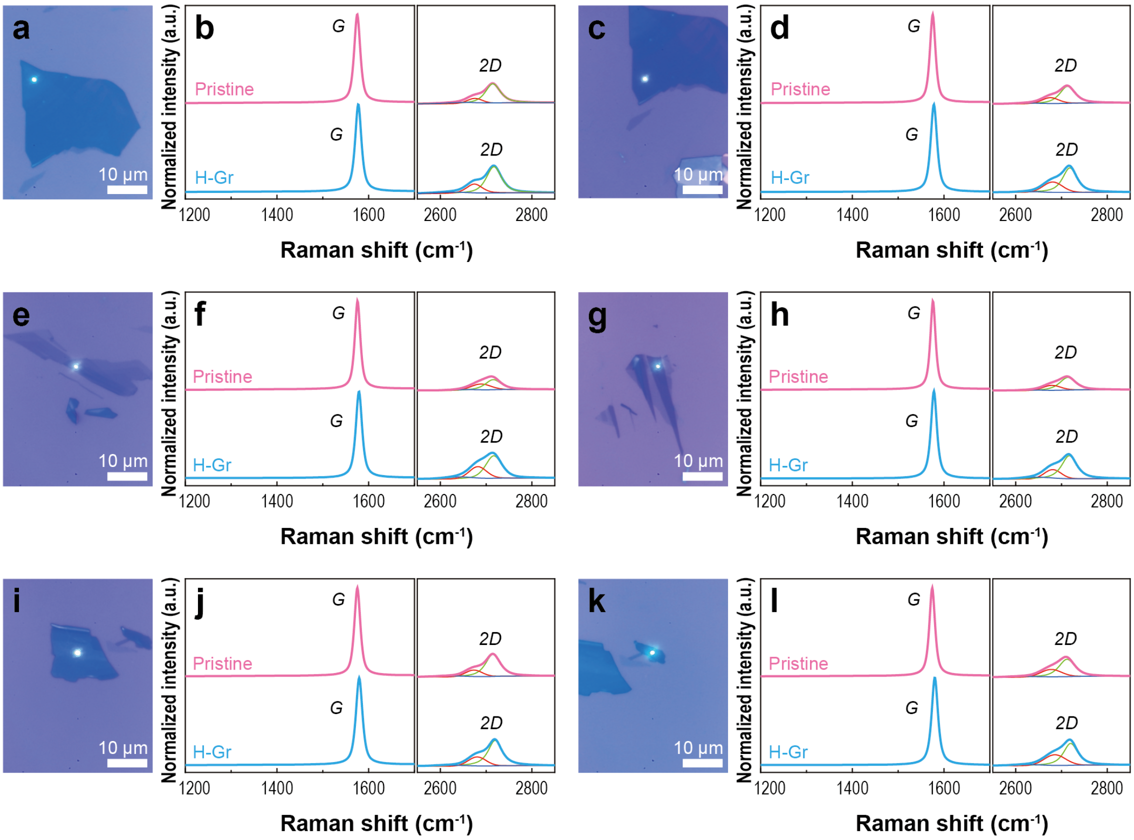

Figure S10. (a), (c), (e), (g), (i), (k) Optical images of laser positions for Raman study at the multilayer graphenes with a uniform surface. (b), (d), (f), (h), (j), (l) Raman spectra of the *G* band and *2D* band of each position before and after exposure to H_2, respectively

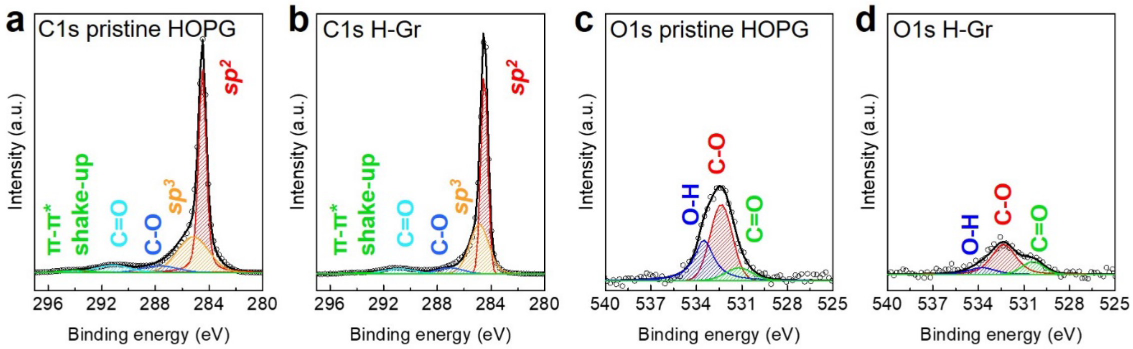

Figure S11. C1s peak of (a) pristine HOPG and (b) H-Gr (hydrogenated HOPG), respectively. O1s spectra of (c) pristine HOPG and (d) H-Gr, respectively

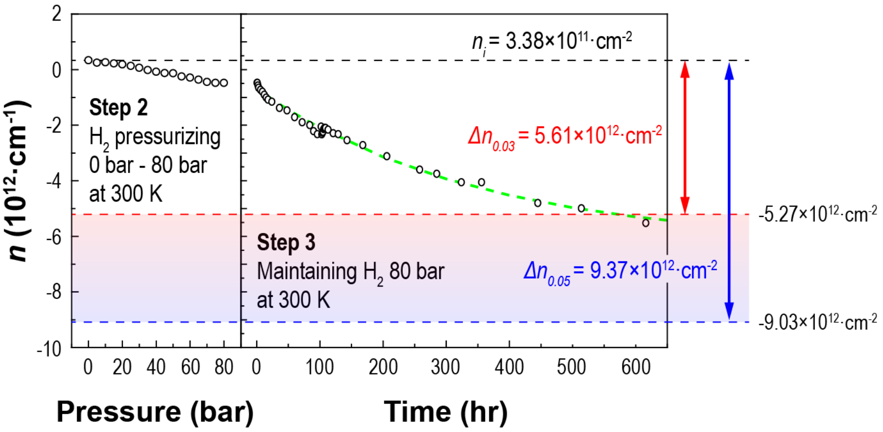

Figure S12. Carrier density (n) as a function of H_2 pressure (step 2: H_2 pressure was applied from 0 bar to 80 bar at 300 K) and of time (step 3: maintaining 80-bar H_2 at 300 K)

We have conducted a detailed quantitative analysis to clarify the relationship between the electron doping concentration and the degree of hydrogenation using XPS analysis and the electrical transfer curve. As shown in Table S1 in the main text, the sp^3-hybridized carbon component increased from 31.26% to 36.19% after hydrogenation, corresponding to an increase of 4.93%. Since sp^3 bonding is considered a direct indicator for C–H covalent bond formation via dissociative hydrogen adsorption, this structural change is closely associated with the electron doping of MLG. The expected increase in carrier density (n) was estimated using the following equations:

$$\Delta n = \Delta N_{sp^3} \times f \quad (1)$$

$$\Delta N_{sp^3} = \Delta N_C \times 0.0493\ (4.93\%) \quad (2)$$

where ΔN_C is the carbon atom density in graphene ($3.8 \times 10^{15}\ cm^{-2}$), and f is the charge transfer fraction. Based on literature, f ranges from 0.03 to 0.05 [S1]. As shown in Figure S12, the experimental n obtained from the transfer curves was $3.38 \times 10^{11}\ cm^{-2}$ (black dotted line) before exposure, reaching $-5.52 \times 10^{12}\ cm^{-2}$ after 616 h at 80 bar of H_2. This experimental value falls within the theoretical range calculated using $f = 0.03$ ($-5.27 \times 10^{12}\ cm^{-2}$, red dotted line) and $f = 0.05$ ($-9.03 \times 10^{12}\ cm^{-2}$, blue dotted line), confirming that 4.93% increase in sp^3 bonding corresponds well with the observed electron carrier density. Hence, the 4.93% of hydrogenation corresponds $-5.52 \times 10^{12}\ cm^{-2}$ of the electron carrier density.

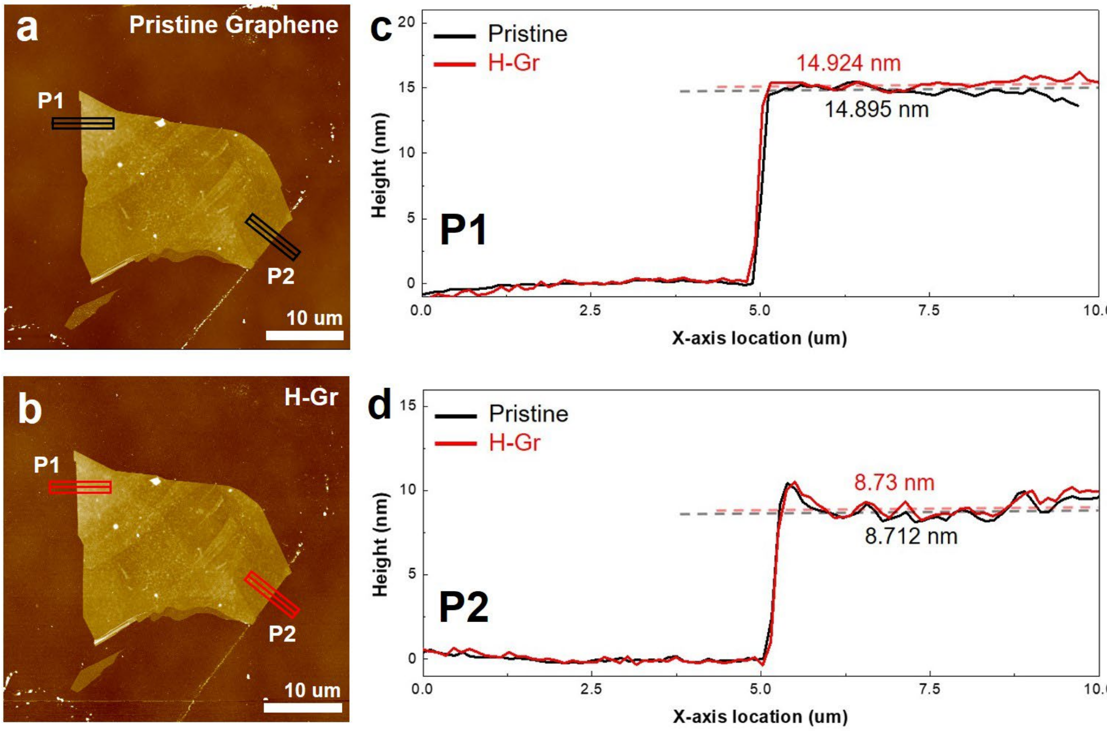

Figure S13. Changes in height of multilayer graphene (a) before (black line) and (b) after (red line) exposure to 80 bar of H_2 pressure. Line profiles obtained at two positions of (c) P1 and (d) P2, marked in (a) and (b), respectively. The heights were the averaged values

To determine the difference in height before and after H_2 exposure, the heights of two points in multilayer graphene were measured using AFM. The average height of H-Gr slightly increased from 14.895 to 14.924 nm at point 1 and from 8.712 to 8.73 nm at point 2; however, the difference was very small.).

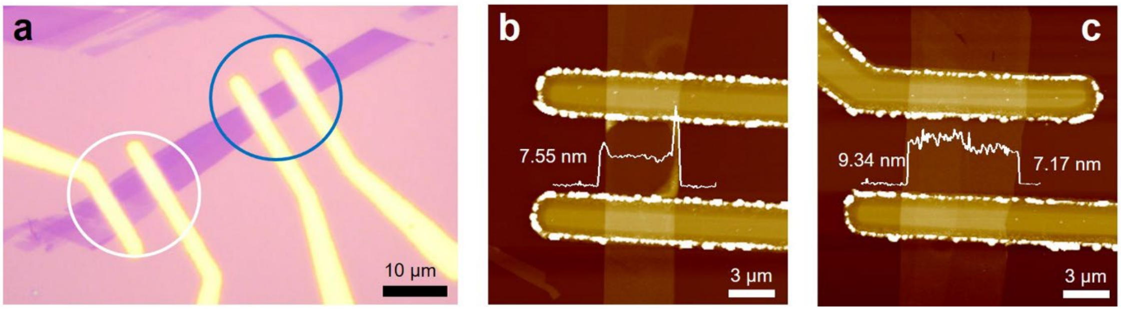

Figure S14. (a) Optical image of s-MLG (blue circle) and MLG-b (white circle). AFM topography of (b) s-MLG and (c) MLG-b. A 2.17-nm height difference was observed in MLG-b

Table S1. XPS results of pristine graphene and H-Gr

Spectrum	Chemical species	Position (eV)		FWHM (eV)		Ratio (%)	
		Pristine	H-Gr	Pristine	H-Gr	Pristine	H-Gr
C1s	π-π* shake-up	293.94	293.62	2.51	2.87	1.64	1.45
	C=O (C=O O-C=O)	291.17	290.86	2.95	2.90	6.72	6.11
	C-O (C-O C-O-C C-OH)	287.70	287.05	3.08	2.39	6.42	5.92
	sp^3	285.19	284.88	2.78	1.70	31.26	36.19
	sp^2	284.50	284.50	0.67	0.61	52.73	49.45
O1s	O-H	533.50	533.77	1.52	2.11	0.45	0.15
	C-O	532.36	532.33	1.71	1.91	0.64	0.56
	C=O	531.19	530.42	2.10	1.70	0.15	0.18

Reference

[S1] Ao Z. M, Hernández-Nieves A. D, Peeters F, et al. Enhanced stability of hydrogen atoms at the graphene/graphane interface of nanoribbons. Appl Phys Lett. 2010;97(23):233109. doi: 10.1063/1.3525377
